# A Novel Regulator Modulates Glucan Production, Cell Aggregation and Biofilm Formation in *Streptococcus sanguinis* SK36

**DOI:** 10.3389/fmicb.2018.01154

**Published:** 2018-05-29

**Authors:** Bin Zhu, Lei Song, Xiangzhen Kong, Lorna C. Macleod, Ping Xu

**Affiliations:** ^1^Philips Institute for Oral Health Research, Virginia Commonwealth University, Richmond, VA, United States; ^2^Department of Microbiology and Immunology, Virginia Commonwealth University, Richmond, VA, United States; ^3^Center for the Study of Biological Complexity, Virginia Commonwealth University, Richmond, VA, United States

**Keywords:** *Streptococcus*, biofilm, transcription factor, glucan, aggregation, CiaR, carbohydrate metabolism

## Abstract

*Streptococcus sanguinis* is an early colonizer of tooth surfaces and a key player in plaque biofilm development. However, the mechanism of biofilm formation of *S. sanguinis* is still unclear. Here, we showed that deletion of a transcription factor, *brpL*, promotes cell aggregation and biofilm formation in *S. sanguinis* SK36. Glucan, a polysaccharide synthesized from sucrose, was over-produced and aggregated in the biofilm of Δ*brpL*, which was necessary for better biofilm formation ability of Δ*brpL*. Quantitative RT-PCR demonstrated that *gtfP* was significantly up-regulated in Δ*brpL*, which increased the productions of water-insoluble and water-soluble glucans. The Δ*brpL*Δ*gtfP* double mutant decreased biofilm formation ability of Δ*brpL* to a level similar like that of Δ*gtfP*. Interestingly, the biofilm of Δ*brpL* had an increased tolerance to ampicillin treatment, which might be due to better biofilm formation ability through the mechanisms of cellular and glucan aggregation. RNA sequencing and quantitative RT-PCR revealed the modulation of a group of genes in Δ*brpL* was mediated by activating the expression of *ciaR*, another *gtfP*-related biofilm formation regulator. Double deletion of *brpL* and *ciaR* decreased biofilm formation ability to the phenotype of a Δ*ciaR* mutant. Additionally, RNA sequencing elucidated a broad range of genes, related to carbohydrate metabolism and uptake, were activated in Δ*brpL*. SSA_0222, a gene involved in the phosphotransferase system, was dramatically up-regulated in Δ*brpL* and essential for *S. sanguinis* survival under our experimental conditions. In summary, *brpL* modulates glucan production, cell aggregation and biofilm formation by regulating the expression of *ciaR* in *S. sanguinis* SK36.

## Introduction

Biofilms are structured, surface-associated communities of microorganisms, which attach to biotic and abiotic surfaces, leading to several acute and chronic health conditions in humans, such as periodontitis (Brouwer et al., 2016; Bergenfelz and Hakansson, 2017; Kreth et al., 2017). The microorganisms in biofilms are encased in a self-produced matrix of hydrated extracellular polymeric substances (EPS) that comprises of polysaccharides, proteins, nucleic acids, and lipids (Flemming and Wingender, 2010). The EPS confers many advantages to microorganisms in response to environmental stresses encountered in the natural and host environments, such as protection of the cells against oxidizing agents, desiccation or the host’s immune defenses. Biofilms also promote horizontal gene transfer by establishing connections between cells that are in close proximity and not fully immobilized (Flemming and Wingender, 2010). Ultimately, due to multiple tolerance mechanisms, bacterial biofilms are highly resistant to antimicrobial therapy and immune clearance, making them extremely effective and persistent invaders and very difficult to eradicate (Davies, 2003).

*Streptococcus sanguinis* (*S. sanguinis*), a Gram-positive facultative anaerobe, exists on tooth surfaces, oral mucosa surfaces and in human saliva (Gross et al., 2012; Francavilla et al., 2014; Seoudi et al., 2015). It does not appear to play a direct role in oral disease, however, it has been reported that oral *S. sanguinis* is frequently the cause of infective endocarditis, a potentially fatal biofilm-associated disease (Bor et al., 2013; Crump et al., 2014). The bacteria can enter the bloodstream via the mouth, gastrointestinal tract or the skin (Duval and Leport, 2008; Holland et al., 2016). Individuals with damage to their heart valve, such as from a congenital heart condition, form ‘vegetation,’ fibrin-platelet complexes, in which the bacteria colonize causing infective endocarditis (Cahill and Prendergast, 2016).

Within the oral cavity, *S. sanguinis* might even be considered a beneficial bacterium with regards to dental caries, as it plays an antagonistic role of competitive exclusion against pathogenic *S. mutans*, depending on the sequence of inoculation (Kreth et al., 2005). However, although thought to be benign, *S, sanguinis* is a pioneering contributor to the biofilm in the human oral cavity known as dental plaque (Socransky et al., 1977; Xu et al., 2011). The adhesion of pioneer bacterial colonizers, such as *S. sanguinis*, to a salivary glycol-protein surface via required anchoring receptors, is essential for the initiation of biofilm development. Thus, *S. sanguinis* may provide a new surface whilst modulating the environment to make it more hospitable for the localization of succeeding microorganisms, which could include pathogens (Maeda et al., 2004).

Hitherto, there are few papers published on the attachment or biofilm formation of *S. sanguinis*. Carbohydrates consumed in the diet are the primary nutrients influencing biofilm formation and sucrose is considered the most cariogenic dietary carbohydrate. Glucosyltransferases (GTFs) are important in sucrose induced plaque formation (Rölla et al., 1985). When using sucrose as the carbon source, GtfP, the only GTF present in *S. sanguinis*, is responsible for glucan synthesis and essential for biofilm formation in *S. sanguinis* (Yoshida et al., 2014; Liu et al., 2017). The overexpression of the *gtfP* gene promotes the production of water-insoluble glucan (WIG) and water-soluble glucan (WSG), which aids biofilm formation (Yoshida et al., 2014; Liu et al., 2017). Transcription of *gtfP* is repressed by an increase in the expression of the arginine (*arg*) biosynthesis gene (Zhu et al., 2017). *Arg* expression is upregulated by the deletion of the *ciaR* gene, part of the CiaH/R two-component system response regulator (CiaR), a well-studied transcription regulator that modulates biofilm formation in *S. sanguinis* (Zhu et al., 2017). It has been shown that a Δ*ciaR* mutant with reduced *gtfP* expression produces less extracellular glucan resulting in deficient biofilm formation (Zhu et al., 2017). Conversely, the deletion of another transcription regulator, *brpT* (Biofilm Regulatory Protein TetR), in *S. sanguinis*, promotes biofilm formation by up-regulating the transcription of *gtfP*, in turn generating more glucan (Liu et al., 2017).

A further important component of the EPS is extracellular DNA (eDNA) for which a role in initial biofilm formation has been firmly established. Whitchurch et al. (2002) demonstrated that the addition of DNase I to the medium of *Pseudomonas aeruginosa* markedly inhibited biofilm initiation in the early stages of growth. Established biofilms were minimally affected (Whitchurch et al., 2002). Furthermore, psl (polysaccharide synthesis locus) transcribes a polysaccharide that can react with eDNA to form a fiber-like web that shapes the biofilm skeleton in *P. aeruginosa* (Wang et al., 2013). In *S. mutans*, eDNA could also cooperate with polysaccharide to impact the early stage of biofilm formation (Castillo Pedraza et al., 2017). However, it is not yet clear whether eDNA contributes to *S. sanguinis* biofilm formation.

Our previous work has constructed a comprehensive mutant library of *S. sanguinis* SK36 (Xu et al., 2011). We performed a high-throughput biofilm assay (unpublished data) in which SSA_0427 was identified as a biofilm-related transcription factor. Genome annotation predicts that the function of coding sequence SSA_0427 is similar to an antibiotic regulatory protein (SARP) family transcription factor in *Streptomyces* (Xu et al., 2007). However, by using amino acid sequence alignment, we propose an alternative function for BrpL as a LuxR family transcriptional regulator. BrpL is highly conserved in *S. sanguinis, S. pyogenes, S. gordonii, S. cristatus, S. dysgalactiae, S. parauberis*, and *S. canis* (Supplementary Figure S1A). No ortholog gene of SSA_0427 was found in *S. mutans* or *S. pneumoniae*. The secondary structure of SSA_0427 was predicted by SMART^1^ (Letunic et al., 2006), to be a classical structure in LuxR family proteins, comprising of a bacterial transcriptional activator domain, a Pfam domain, three TRP domains and a tetratricopeptide repeat (Supplementary Figure S1B).

In this study, we showed that the deletion of the SSA_0427 gene was detrimental to biofilm formation in *S. sanguinis* SK36. As a result, it was named *brpL* (Biofilm Regulatory Protein LuxR). We used confocal laser scanning microscopy (CLSM), quantitative RT-PCR (qRT-PCR) and functional assays to characterize the role of *brpL* in the regulation of glucan production, cell aggregation and biofilm formation. RNA sequencing (RNA-seq) data revealed that *ciaR* mediated the elevation of biofilm formation in Δ*brpL*.

## Results

### Deletion of *brpL* in *S. sanguinis* SK36 Increases Cell-Surface Attachment Strength

In our previous work, a comprehensive mutant library of *S. sanguinis* SK36 was generated by high-throughput PCR (Xu et al., 2011). Firstly, we analyzed biofilm formation of predicted transcriptional regulator mutants by the growth of biofilms plated on polystyrene microtiter plates overnight under microaerobic conditions. All strains were grown in BM media supplemented with 1% sucrose and biofilms examined using CV staining. Our initial screening indicated that deletion of the *brpL* gene resulted in an increased biofilm phenotype in comparison to the wild-type SK36 (WT) (data not shown). To validate whether *brpL* was a biofilm related gene, we recorded growth curves of Δ*brpL* and WT in BM supplemented with 1% sucrose. More rapid growth of the Δ*brpL* mutant biofilm in comparison to the WT was observed in early log phase although a similar cell density was observed at stationary phase (Supplementary Figure S2A). Due to bacterial cell aggregation, colony forming units (CFU) could not be counted accurately. WT and Δ*brpL* strains were grown in BM supplemented with 1% sucrose for 7.5 h. The cells were then harvested, stained by crystal violet (CV) and biomass was recorded by digital pictures (Supplementary Figure S2B). It was observed that Δ*brpL* accumulated more biomass than WT, indicating a faster growth rate of Δ*brpL*. More severe aggregation appeared in Δ*brpL* at late log phase, which increased the standard deviations of the OD_600_ values (Supplementary Figure S2A). Growth curve comparison could not exclude the possibility that better biofilm formation of Δ*brpL* was caused by increased growth rate.

**FIGURE 1:**
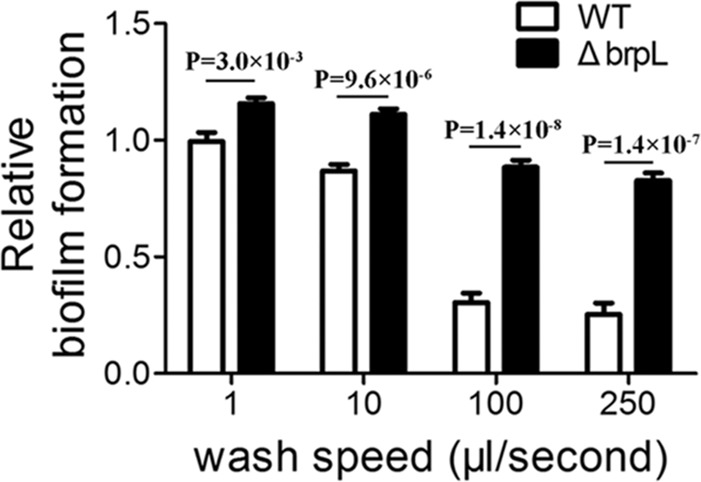
The biofilm attachment of WT and Δ*brpL* on polystyrene microtiter plates. The 1-day biofilms of WT and Δ*brpL* were quantified by CV staining. The washing steps were done by using a Caliper Sciclone G3 liquid handling robot with different washing speeds. *P*-values were generated by Student’s *t-*test. Means and standard deviations from triplicate experiments are shown.

To further explore the precise mechanism for improved biofilm phenotype in the Δ*brpL* deletion mutant, the cell-surface attachment of the biofilms was investigated. Resulting biofilms from the growth assay were washed using a Caliper Sciclone G3 liquid handling robot (PerkinElmer, United States) which generated different speeds of washing flow. A higher magnitude of difference was seen after a severe wash (**Figure 1**), which suggested that the Δ*brpL* mutant had better attachment ability to the surface of polystyrene plates (**Figure 1**).

### The Biofilm of Δ*brpL* Contains More Biomass and Cell Aggregation

**FIGURE 2:**
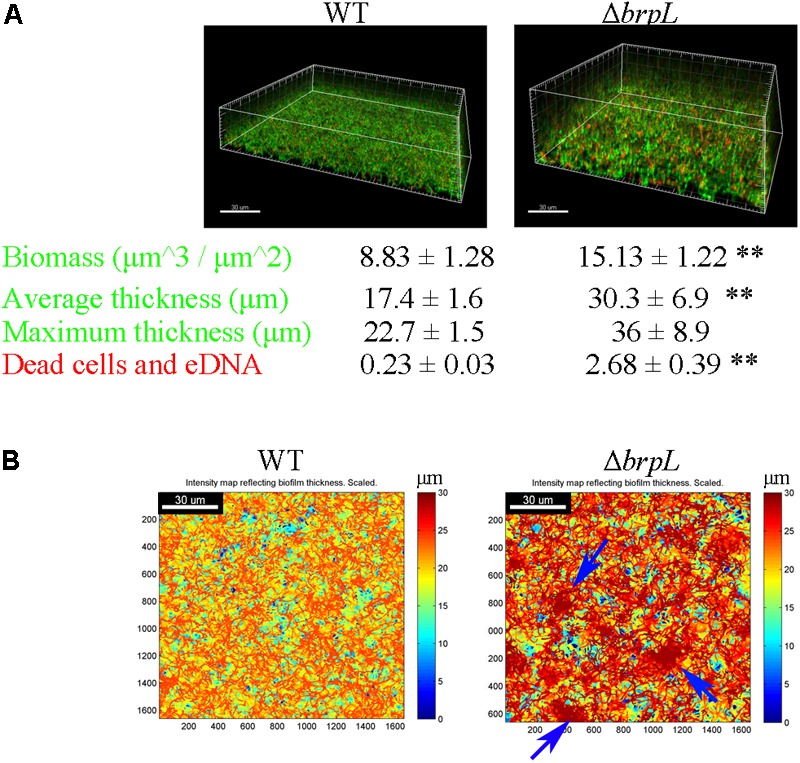
The characteristic of biomass and cell distribution in the biofilms of WT and Δ*brpL.* **(A)** The biofilms of WT and Δ*brpL* were cultured in a 4-well chamber for 24 h, stained by SYTO 9 (green)/PI (red) and captured by CLSM. 3D architectures of biofilms were shown on the top. Biomass (the signal of SYTO 9) and PI signal representing dead cells and eDNA in CLSM images were calculated by COMSTAT analysis (mean ± SD). Average and maximum thickness were quantified by COMSTAT based on the signal of SYTO 9 (mean ± SD). **(B)** Heat maps showing the thickness of biofilms (overlap of biomass in all slices) were made by COMSTAT analysis, which reflected the distribution of biomass in biofilms. Blue arrows point to cell aggregation in the biofilm of Δ*brpL*. Scale bars were indicated on the corresponding images. All the data in **(A)** are compared with their WT control. ^∗∗^*P* ≤ 0.01, Student’s *t-*test. Means and standard deviations from triplicate experiments are shown.

The structure of biofilm (thickness) and microbial characteristics (biomass and live/dead ratio) were investigated using CLSM and quantified using a COMSTAT script in Matlab software (Heydorn et al., 2000). Briefly, biofilms of WT and Δ*brpL* were cultured in a 4-well chamber for 24 h and then treated with SYTO9 to mark live cells and propidium iodide (PI) to mark dead cells and eDNA. In comparison to WT biofilm, the biofilm of Δ*brpL* was approximately twice as thick (WT: 17.4 ± 1.6 μm, Δ*brpL*: 30.3 ± 6.9 μm) and formed nearly twice as much biomass (WT: 8.83 ± 1.28 μm^3^/μm^2^, Δ*brpL*: 15.13 ± 1.22 μm^3^/μm^2^) (**Figure 2A**). This result demonstrated a superior biofilm formation ability of Δ*brpL* in comparison to WT, which was consistent with the finding of increased attachment ability (**Figure 1**). Moreover, a significantly stronger PI (red) signal appeared in the biofilm of Δ*brpL*, indicating a larger amount of dead cells and eDNA (**Figure 2A**).

**Figure 2B** shows intensity maps of WT and mutant strain biofilms, which illustrates through color change, differences in biofilm thickness. The images were generated by Matlab script, COMSTAT (Heydorn et al., 2000). Cells were uniformly distributed in the biofilm of WT, but markedly aggregated in that of Δ*brpL* (**Figure 2B**). An observation made but not quantified, was that cell aggregation accumulated at upper layers of the Δ*brpL* biofilm and a cell cavity formed in the layers under a cell aggregate (Supplementary Figure S3). Cell aggregation could also be seen when strains were cultured in BM supplemented with 1% sucrose under shaking conditions (200 rpm) in 14 mL tubes (Supplementary Figure S4A). Additionally, many macrocolonies formed on the surface of a bacteriological petri dish in BM supplemented with 1% sucrose, providing further evidence of aggregation of the Δ*brpL* mutant (Supplementary Figure S4B).

### Cell Aggregation Is Not Caused by eDNA in Δ*brpL*

Only very little PI signal (red) was seen in the biofilm of WT in comparison to Δ*brpL* (**Figure 2A**). In fact, most of red signal have a cell-like shape, indicating they were dead cells rather than eDNA (Supplementary Figure S3). No eDNA signal smear was observed in the image (Supplementary Figure S3). These data implied that cell aggregation might not be promoted by a larger amount of eDNA. To further confirm this hypothesis, we treated the biofilms with 100 U/mL of DNase I which non-specifically cleaves eDNA. DNase I treatment did not result in any significant difference in biofilm formation, which suggested that eDNA did not impact biofilm formation under our experimental conditions (Supplementary Figure S5).

### Dense Fiber-Like Matrix Exists in the Biofilm of Δ*brpL*

**FIGURE 3:**
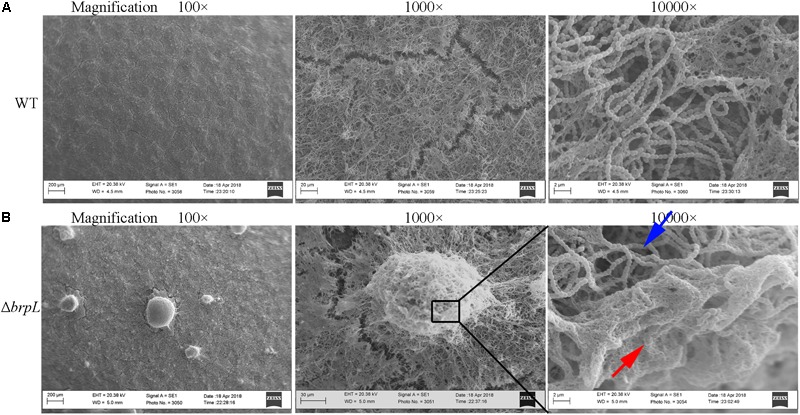
The fiber-like matrix in the biofilms of WT and Δ*brpL*. The 1-day biofilms of WT **(A)** and Δ*brpL* **(B)** were captured by SEM with magnifications of 100×, 1000×, and 10000×, respectively. Scale bars were shown on each image. The red arrow indicates fiber-like matrix at the periphery of cell aggregation and the blue arrow points to cells inside of the aggregate.

To confirm cells aggregated in the biofilm of Δ*brpL*, biofilms were observed by scanning electron microscopy (SEM), which showed a similar phenomenon that much more cell aggregation appeared on the surface of Δ*brpL* biofilm than that of WT (**Figure 3**, 100-fold magnified figures). Fiber-like matrix existed on the surface of both WT but seemingly more abundant in the biofilm of Δ*brpL* (**Figure 3**). A larger amount of fiber-like matrix linked cells together, which might lead to a better biofilm formation ability of Δ*brpL*. The 10000-fold magnified figure showed an interesting result that the periphery of cell aggregation in the biofilm of Δ*brpL* was fully covered by a layer of fiber-like matrix (**Figure 3B**, red arrow). However, much less fiber-like matrix was observed in the center of the cell aggregate (**Figure 3B**, blue arrow). We put forward a theory that the cells closer to the periphery of the cellular aggregate are also closer to any nutrients which would facilitate the synthesis of fiber-like matrix.

### The Deletion of *brpL* Promotes Polysaccharide Production and Aggregation

**FIGURE 4:**
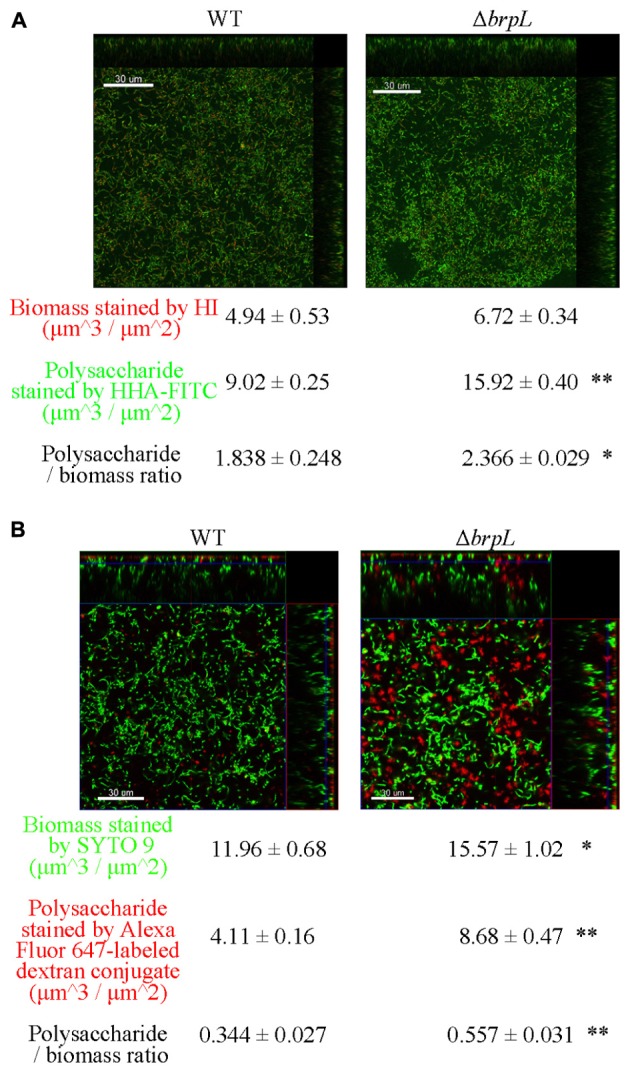
The distribution of polysaccharide in the biofilms of WT and Δ*brpL*. The 1-day biofilms were cultured in 4-well chambers for 24 h. **(A)** Biofilms were stained by HHA-FITC (green)/HI (red), which marked polysaccharide with mannosyl units and cells, respectively. **(B)** Cells in biofilms were stained by SYTO 9 (green) and polysaccharide with poly-(α-D-1, 6-glucose) linkages was marked by Alexa Fluor 647-labeled dextran conjugate (red). CLSM images were shown. Scale bars were indicated on the corresponding images. Biomass, polysaccharide production and biomass/polysaccharide ratio were calculated by COMSTAT analysis (mean ± SD). All the data in **(A,B)** are compared with their WT control. ^∗^*P* ≤ 0.05, ^∗∗^*P* ≤ 0.01, Student’s *t-*test. Means and standard deviations from triplicate experiments are shown.

A previous study illustrated that filamentous structures were related to the production of glucan in Δ*brpT* (Liu et al., 2017). In addition, the phenomenon of cell aggregation has been associated with the over-production of extracellular polysaccharide in other bacteria (Clark and Gibbons, 1977; McNab and Jenkinson, 1992; Zhu et al., 2016). To quantify the amount of polysaccharide, biofilms were grown in 4-well chambers for 24 h. Two methods were used to stain cells and polysaccharide. In **Figure 4A**, cells were identified by Hexidium iodide (HI) and polysaccharide, containing α-(1, 3) or α-(1, 6) linked mannosyl units, was stained by *Hippeastrum* hybrid lectin (HHA)-FITC (Ma et al., 2009). In **Figure 4B**, cells were marked by SYTO 9 and polysaccharide, with poly-(α-D-1, 6-glucose) linkages, was identified by Alexa Fluor 647-labeled dextran conjugate (Koo et al., 2010). Images were taken by CLSM and quantified using a COMSTAT script in Matlab (Heydorn et al., 2000). Both staining methods showed that the ratio of polysaccharide/biomass of Δ*brpL* was greater than that of WT, which suggested Δ*brpL* had better polysaccharide production ability (**Figure 4**). The signal of polysaccharide with mannosyl units overlapped with cells (**Figure 4A**), indicating that this kind of polysaccharide existed on cell surface or inside of cells. However, the polysaccharide with α-(1, 6) linked glucosyl units localized in the gaps between cells (**Figure 4B**), which might indicate component of fiber-like matrix.

### *gtfP* Is Important for Δ*brpL* to Impact Biofilm Formation

**FIGURE 5:**
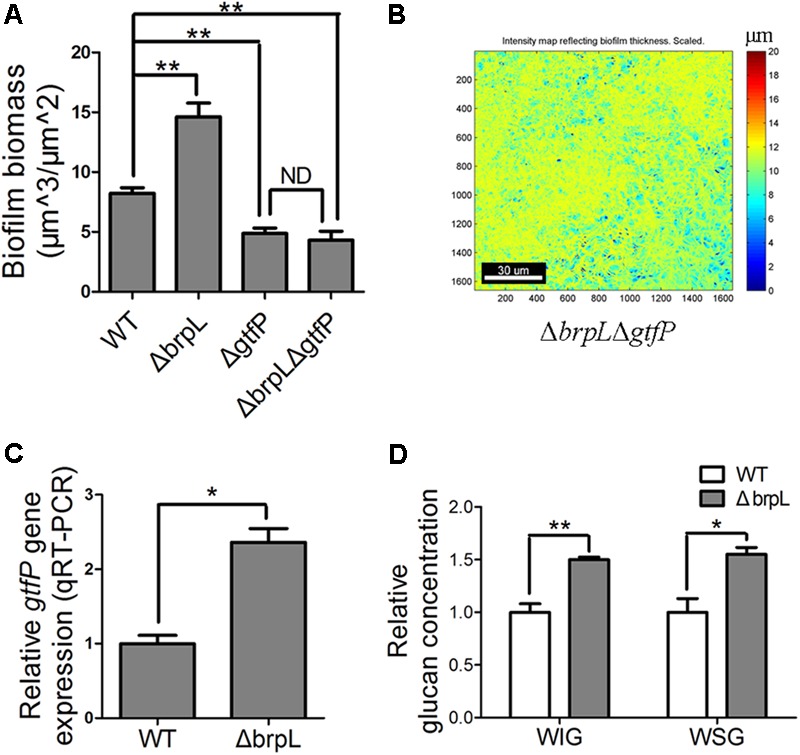
The *gtfP* gene is important for Δ*brpL* to regulate biofilm formation. **(A)** The biofilms were stained by SYTO 9 (green)/PI (red), visualized by CLSM and quantified by COMSTAT. Biofilm biomass was shown. **(B)** The heat map of biomass distribution in the biofilm of Δ*brpL*Δ*gtfP* was made by COMSTAT analysis. A scale bar was shown on the image. **(C)** qRT-PCR was performed to examine the expression of *gtfP* gene in Δ*brpL*. **(D)** The WIG and WSG of WT and Δ*brpL* were measured by the method described in section “Materials and Methods.” ^∗^*P* ≤ 0.05, ^∗∗^*P* ≤ 0.01, Student’s *t-*test. Means and standard deviations from triplicate experiments are shown. ND indicates no significant difference.

Glucan, a polysaccharide of D-glucose monomers, is one of the most important polysaccharide for biofilm formation of *S. sanguinis* (Yoshida et al., 2014; Liu et al., 2017). GtfP, the only GTF in *S. Sanguinis*, is essential for glucan production (Yoshida et al., 2014; Liu et al., 2017). To understand the role of glucan in the biofilm of Δ*brpL*, a Δ*brpL*Δ*gtfP* double mutant was constructed and the biofilm biomass was stained by SYTO 9/PI and observed by CLSM. The biomass of Δ*gtfP* was significantly (*P* ≤ 0.01) less than WT (**Figure 5A** and Supplementary Figure S6). The Δ*brpL*Δ*gtf* double decreased biomass to show a similar phenotype to a Δ*gtfP* mutant (**Figure 5A** and Supplementary Figure S6). Furthermore, the Δ*brpL*Δ*gtfP* mutant could not promote cell aggregation (**Figure 5B**). These data suggested the effects of the *brpL* deletion on biofilm formation were solely through its ability to regulate *gtfP* expression and glucan was essential for cell aggregation.

As the polysaccharide with α-(1, 6) linked glucosyl units was over-produced in Δ*brpL* (**Figure 4B**), we hypothesized that *gtfP*, might be regulated by *brpL*. We quantified the transcription of *gtfP* by qRT-PCR, which illustrated that *gtfP* was significantly activated in Δ*brpL* (**Figure 5C**). It has been demonstrated that GtfP is responsible for the generation of WIG and WSG (Yoshida et al., 2014; Liu et al., 2017). As a result, the concentrations of WIG and WSG were measured. The biofilm of Δ*brpL* contained more WIG and WSG, which further confirmed that glucan was over-produced in Δ*brpL* (**Figure 5D**). Glucan may be one of the essential components in the fiber-like matrix and may facilitate cell aggregation.

### Cell Cavities Observed at the Location of Glucan Aggregation in the Biofilm of Δ*brpL*

**FIGURE 6:**
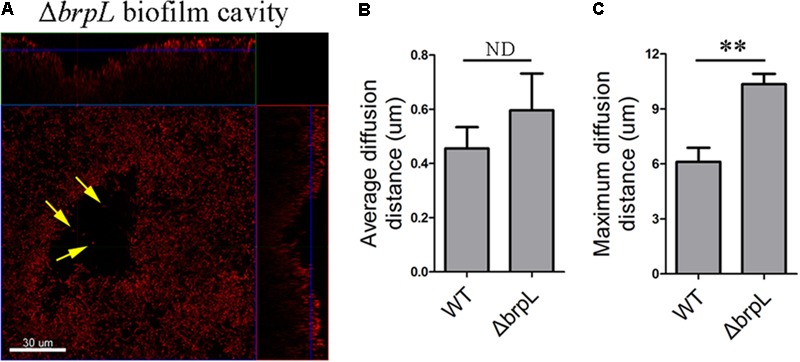
A biofilm cavity and single cells inside of the cavity. **(A)** The 1-day biofilm of Δ*brpL* was stained by HI and then captured by CLSM. Yellow arrows point to single cells existed inside of the biofilm cavity. A scale bar was shown on the image. Based on the SYTO 9 signal of CLSM images in **Figure 2A**, average **(B)** and maximum **(C)** diffusion distances were measured by COMSTAT analysis to simulate the distance from nutrient to cells. ^∗∗^*P* ≤ 0.01, Student’s *t-*test. ND indicates no significant difference. Means and standard deviations from triplicate experiments are shown.

As illustrated in Supplementary Figure S3, cell cavities were observed in the biofilm of Δ*brpL*. A similar phenomenon was well-studied in *P. aeruginosa* which forms mushroom-like three-dimensional microcolonies (Stoodley et al., 2002; Ma et al., 2009). Psl polysaccharide is distributed on the periphery of these microcolonies. Programed cell death generated cavities in the centers of microcolonies (Ma et al., 2009). Some planktonic cells live in these cavities for seeding dispersal (Ma et al., 2009). In the biofilm of Δ*brpL*, a small number of single living cells were observed within cavity boundaries (**Figure 6A**). However, unlike *P.aeruginosa, S. sanguinis* SK36 is a non-motile bacterium, therefore it is uncertain whether these single cells were participating in seeding dispersal in *S. sanguinis*.

To examine whether or not cell aggregation was the cause of a cell cavity, we calculated the diffusion distance of nutrient to cells based on CLSM images in **Figure 2** by using the COMSTAT script (Heydorn et al., 2000). For example, the maximum diffusion distance is the longest distance from the periphery of a microcolony to its center. The average diffusion distance is the average value from the periphery to every single cell. We found that although the average diffusion distances between WT and Δ*brpL* were not significantly different, the Δ*brpL* mutant had a significantly larger maximum diffusion distance (*P*-value ≤ 0.01) (**Figures 6B,C**). This would suggest a substantial proportion of nutrients may diffuse a much further distance to reach the cells at the center of an aggregate within a Δ*brpL* biofilm. We hypothesize that the nutrients may be consumed by cells in closer proximity to the aggregate periphery, resulting in nutrient deficit of the inner cells and hence cell death. Additionally, a longer diffusion distance could also lead to the accumulation of harmful metabolites, such as acid or H_2_O_2_, which would to kill cells at the center of the cell aggregate.

### The Deletion of Δ*brpL* Increased the Tolerance of Biofilm to Ampicillin Treatment

It has been widely reported that the aggregation of bacteria into EPS-coated biofilm is associated with increased antibiotic resistance (Davies, 2003). Polysaccharide is one of the most important components of EPS and contributes to antibiotic resistance (Flemming and Wingender, 2010). It is conceivable that cells inside of an aggregation might be protected by peripheral cells and polysaccharide from antibiotics attack in the biofilm of Δ*brpL*. We treated planktonic cells and biofilms by series concentrations of ampicillin for 2 h, respectively. The tolerance of Δ*brpL* was the same as that of WT in planktonic cells but eight times higher than the WT in the biofilm population (**Table 1**). This suggested that the tolerance to ampicillin treatment was increased by better biofilm formation ability in Δ*brpL* (**Table 1**). Cell aggregation may be essential for this increased tolerance.

**Table 1:** Maximum tolerance concentration of strains to ampicillin.

Strain name	Maximum tolerance concentration (μg/mL)	
	Biofilm	Planktonic
SK_36	0.625	0.25
SSA_0427	5	0.25

### Δ*brpL* Regulates a Group of Genes Through Promoting the Expression of *ciaR*

**FIGURE 7:**
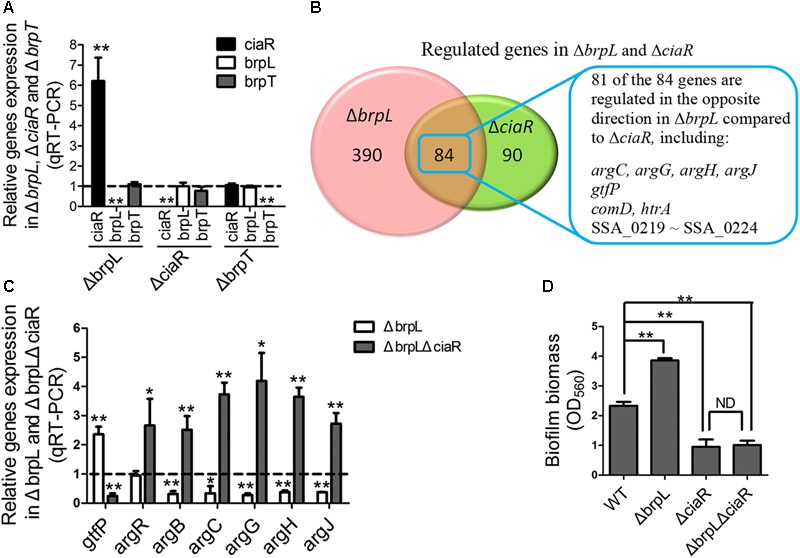
BrpL controls biofilm formation via a downstream regulator CiaR. **(A)** qRT-PCR was performed to examine the relationships of regulation between *ciaR, brpL*, and *brpT*. **(B)** Based on RNA-seq data (Supplementary Datasheet 1), the number of overlapped genes with differential expression (fold change ≥ 1.5 or ≤ 0.667 and *p*-value ≤ 0.05) in Δ*brpL* and Δ*ciaR* was shown by Venn diagram. **(C)** The expression of genes in WT, Δ*brpL* and Δ*brpL*Δ*ciaR* was tested by qRT-PCR. All of the data were relative to their WT controls. **(D)** The biofilm biomass of strains was measured by CV staining. ^∗^*P* ≤ 0.05, ^∗∗^*P* ≤ 0.01, Student’s *t-*test. Means and standard deviations from triplicate experiments are shown.

As mentioned above, previous works show that a response regulator, CiaR, of the CiaRH two-component system and a transcriptional regulator, BrpT, impact biofilm formation by affecting glucan production, which appears to be similar to *brpL* (Liu et al., 2017; Zhu et al., 2017). By using qRT-PCR, we tested the relationships between these three regulators. The only relationship was that the transcription of the *ciaR* gene was significantly increased in Δ*brpL* (**Figure 7A**). To further explore the relationship between *brpL* and *ciaR*, RNA-seq of Δ*brpL* was executed. Consistent with the qRT-PCR result, the expression of the *ciaR* gene was fivefold increased in Δ*brpL* (Supplementary Datasheet 1). Furthermore, there were 474 genes significantly modulated (fold change ≥ 1.5 or ≤ 0.667 and *P*-value ≤ 0.05 in Δ*brpL*), within which 84 genes were also regulated in Δ*ciaR* (**Figure 7B**). Surprisingly, 81 of the 84 overlapped genes were regulated in the opposite direction in Δ*brpL* compared to Δ*ciaR*, including genes associated with arginine biosynthesis (*argC, argG, argH*, and *argJ*), glucan production (*gtfP*) and cell competence (*comD* and *htrA*) (**Figure 7B**). These data suggested that BrpL repressed the expression of *ciaR* in *S. sanguinis* SK36.

Furthermore, qRT-PCR results confirmed that *argB, argC, argG, argH*, and *argJ* were all significantly down-regulated in Δ*brpL* (**Figure 7C**), which was in contrast to the upregulation of these genes seen in the Δ*ciaR* mutant (Zhu et al., 2017). In addition, a Δ*brpL*Δ*ciaR* double genes deletion mutant resulted in a phenotype similar to that of the Δ*ciaR* where the *arg* genes were all up-regulated (**Figure 7C**) (Zhu et al., 2017). Similarly, the expression of *gtfP* was promoted in Δ*brpL*, while suppressed in both Δ*ciaR* and Δ*brpL*Δ*ciaR* (**Figure 7C**) (Zhu et al., 2017). These results further confirmed that *brpL* modulated *gtfP* and the *arg* genes through the regulation of *ciaR*.

### Δ*brpL* Modulates Biofilm Formation Through the Downstream Regulator *ciaR*

Our previous work reveals that by promoting the expression of arginine biosynthetic genes, particularly the *argB* gene, the *ciaR* mutation reduces the expression of *gtfP*, resulting in a decreased glucan production and the formation of a fragile biofilm in *S. sanguinis* (Zhu et al., 2017). Additionally, the Δ*argB* mutant exhibited severe autoaggregation (Zhu et al., 2017). The transcription level of the *argB* gene was low in the Δ*brpL* mutant (**Figure 7C**), which might contribute to the cell aggregation phenotype in Δ*brpL* (**Figures 2B, 3** and Supplementary Figure S4). We hypothesized that the activation of *ciaR* might facilitate Δ*brpL* in enhancing biofilm formation and aggregation. To this end, we showed that the Δ*brpL*Δ*ciaR* double mutant was deficient in biofilm formation and accumulated less cell aggregation (**Figure 7D** and Supplementary Figure S4B). Together, these data indicated that *ciaR* played an essential role in Δ*brpL* to affect biofilm formation.

CiaH is the histidine kinase of the CiaH/R two-component system, which senses a stimulus and transfers a signal to the response regulator *ciaR* (Zähner et al., 2002). Previous works demonstrate that *ciaH* modulates biofilm formation in *S. pneumoniae* (Zähner et al., 2002). Here, we showed the deletion of *ciaH* also reduced biofilm formation in *S. sanguinis* (Supplementary Figure S7A). Conversely, the expression of *ciaH* was increased in Δ*brpL*, which implied that both *ciaH* and *ciaR* participated in the network modulated by *brpL* (Supplementary Figure S7B). We propose a certain stimulus might be generated by the deletion of *brpL*, sensed by *ciaH* and responded to by *ciaR* to change biofilm formation in *S. sanguinis*.

Collectively, the above data suggested that Δ*brpL* modulated biofilm formation through the downstream regulator *ciaR*.

### A Broad Range of Carbohydrate Metabolism Pathways Are Controlled by Δ*brpL*

**FIGURE 8:**
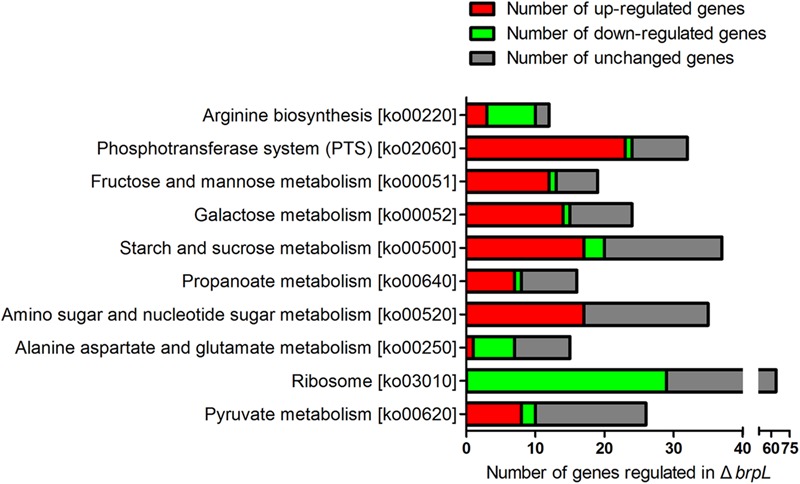
Numbers of differential expressed genes involved in each pathway in Δ*brpL*. The names of KEGG pathways and their relative ko numbers (originated from KEGG database) were listed in y-title of the figure. Differential expressed genes (fold change ≥ 1.5 or ≤0.667 and *p*-value ≤ 0.05) were classified into different pathways. The numbers of regulated genes were counted by RPAS script running in Matlab software.

To further analyze the RNA-seq data of Δ*brpL*, we created a Matlab script to count numbers of regulated genes in each pathway (Supplementary Script of Matlab). The pathway information was obtained from the KEGG database (Kanehisa et al., 2004). We listed the numbers of up- and down-regulated genes, total genes and the ratio of regulated genes/total genes in each pathway (Supplementary Datasheet 2). Pathways were ranked by the ratio of regulated genes/total genes from large to small and top 10 most influenced pathways were exhibited. The data illustrated that a broad range of carbohydrate metabolism pathways were impacted by the deletion of *brpL*, including fructose and mannose metabolism, galactose metabolism, starch and sucrose metabolism, amino sugar and nucleotide sugar metabolism, alanine, aspartate and glutamate metabolism and pyruvate metabolism (**Figure 8**). Most of genes involved in these pathways were upregulated except for genes in the alanine, aspartate and glutamate metabolism pathway (**Figure 8**), which implied that carbohydrate metabolism might be enhanced in Δ*brpL*. Additionally, a large amount of phosphotransferase system (PTS) genes were up-regulated (**Figure 8**), indicating that the uptake of carbohydrate might be promoted in Δ*brpL* (Kotrba et al., 2001). The increased uptake and metabolism of carbohydrate might elevate the intracellular concentration of carbon sources, which in turn would have supported polysaccharide production and biofilm formation. These results were consistent with the finding that two kinds of polysaccharide were over-producted in Δ*brpL* (**Figure 4**).

### A PTS Gene SSA_0222 Is Essential for the Growth of *S. sanguinis*

**FIGURE 9:**
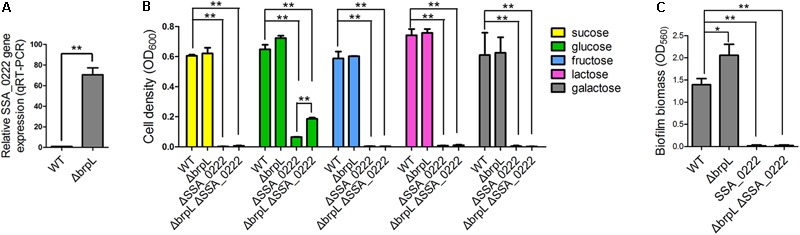
The effect of SSA_0222 on growth and biofilm formation. **(A)** The expression of SSA_0222 in WT and Δ*brpL* was tested by qRT-PCR. The relative expression of SSA_0222 was shown. **(B)** Strains were cultured in BM supplemented with different kinds of carbohydrate for 24 h. Cell growth was monitored at 600 nm with a plate reader. **(C)** The 1-day biofilms of strains were quantified by CV staining. All of the data were relative to their WT controls. ^∗^*P* ≤ 0.05, ^∗∗^*P* ≤ 0.01, Student’s *t-*test. Means and standard deviations from triplicate experiments are shown.

RNA-seq data revealed that a gene cluster (SSA_0219, SSA_0220, SSA_0221, SSA_0222, and SSA_0224) showed high fold upregulation in Δ*brpL* (fold change > 110 for all of the five genes) and relative downregulation in Δ*ciaR* (fold change < 0.25 for all of the five genes) (**Figure 7B**, Supplementary Figure S8 and Supplementary Datasheet 1). The qRT-PCR results confirmed SSA_0222 was over-expressed in Δ*brpL* (**Figure 9A**). Genome annotation predicts that SSA_0219, SSA_0220, SSA_0221, and SSA_0222 were PTS system mannose-specific transporter subunits, while the function of SSA_0224 was unknown (Supplementary Datasheet 1). Since PTS genes are related to the uptake of carbohydrate (Kotrba et al., 2001), we tested the growth of these PTS gene mutants in BM supplemented with five kinds of carbon sources: sucrose, glucose, fructose, lactose and galactose. The deletion of SSA_0219, SSA_0220, and SSA_0221 had no impact on cell growth or biofilm formation (data not shown). Surprisingly, the SSA_0222 mutant had only minimal growth in BM supplemented with glucose and failed to grow in all other media (**Figure 9B**). Although it remains unclear if SSA_0222 had a direct impact on carbohydrate uptake, under our experimental conditions, this gene was essential for the survival of *S. sanguinis*. The biofilm formation of ΔSSA_0222 was severely decreased which could have been caused by the growth deficiency of the bacteria (**Figure 9C**).

As SSA_0222 was dramatically up-regulated in Δ*brpL*, we proposed that SSA_0222 was involved in the influence of *brpL* biofilm formation control. To this end, a Δ*brpL*ΔSSA_0222 double gene deletion mutant was constructed. The mutant showed similar characteristics to a ΔSSA_0222 mutant in growth and biofilm formation (**Figures 9B,C**). Since the growth of Δ*brpL*ΔSSA_0222 was severely inhibited, it was uncertain whether SSA_0222 contributed to an increased biofilm formation in Δ*brpL*. However, Δ*brpL* grew faster than WT at log phase (Supplementary Figure S2), indicating that an over-expression of SSA_0222 might promote carbohydrate uptake and as a result supply more carbon source for the biofilm formation of Δ*brpL*. Further studies are required to elucidate the precise function of SSA_0222 and its relationship with BrpL as these initial findings suggest importance in biofilm regulation.

It was interesting to observe that Δ*brpL*ΔSSA_0222 grew better than ΔSSA_0222 in BM supplemented with 1% glucose, indicating that the expression of other genes might be altered in Δ*brpL* to promote the uptake and/or metabolism of glucose in Δ*brpL* (**Figure 9B**). This result was consistent with the RNA-seq data that a broad range of genes related to carbohydrate metabolism and uptake were activated in Δ*brpL* (**Figure 8**).

## Discussion

**FIGURE 10:**
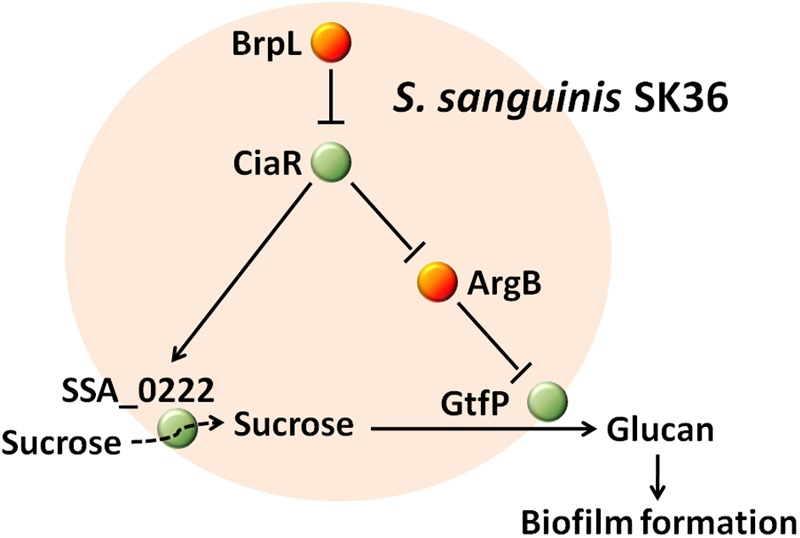
The mechanism by which BrpL modulates biofilm formation in *Streptococcus sanguinis* SK36. GtfP, a glucosyltransferase, is responsible for glucan production. SSA_0222, a component of a PTS, may play an important role in the uptake of sucrose and is essential for survival in BM supplemented with 1% sucrose. BrpL regulates the expression of *gtfP* and SSA_0222 through the inhibition of another biofilm-related regulator *ciaR* and as a result controls the biofilm formation of *S. sanguinis* SK36.

The effectiveness of *S. sanguinis*, as a pioneering organism, to form attachments to surfaces within the oral cavity, could be attributed to particular cell-surface attachment traits and/or particular initial biofilm properties (Socransky et al., 1977; Maeda et al., 2004). As *S. sanguinis* lays the foundations for biofilm that may lead to disease-contributing plaque formation, it is essential to identify and characterize biofilm related genes to aid in developing novel therapeutic agents against oral disease. In this study, we present coding sequence SSA_0427 as a new LuxR family transcription regulator that controls biofilm formation by modulating glucan production and may also function to increase sucrose uptake through SSA_0222 (**Figure 10**). The deletion of SSA_0427, which we have called *brpL*, resulted in a mutant with a higher tolerance to ampicillin exposure and better cell-surface attachment ability to prevent it from being washed away (**Table 1** and **Figure 1**). These characteristics would both be beneficial for maintenance of *S. sanguinis* in oral cavity in the presence of sucrose as carbon source. Cell and glucan aggregation formed in the biofilm of Δ*brpL* may be important factors leading to these phenotypes (Flemming and Wingender, 2010; Hobley et al., 2015). However, it is still not clear about whether or when the expression of *brpL* is decreased to improve the biofilm formation of *S. sanguinis*. To reduce the biofilm formation, further studies on what influences the activation of *brpL* expression need to be performed in future.

Notwithstanding, the current research on *brpL* helped us elucidate an interesting PTS gene SSA_0222 that is essential for the uptake of carbon sources and further confirm the mechanisms by which CiaR modulates biofilm formation. SSA_0222 and CiaR may be better targets to decrease biofilm formation of *S. sanguinis*.

*Streptococcus sanguinis* SK36 is non-motile and cells are not assembled by a proactive movement which raises the question of how the cells aggregate. A previous work demonstrated that localized cell death focuses mechanical forces during biofilm development (Asally et al., 2012). These forces promote the self-assembly of a wrinkle structure (Asally et al., 2012), which could explain the phenomenon of aggregation in *S. sanguinis*. However, it is certain that polysaccharide is an essential skeleton for bacteria to produce biofilms with a complex three-dimensional structure (Flemming and Wingender, 2010; Wang et al., 2013; Hobley et al., 2015). In *S. sanguinis*, the biofilm formed by Δ*gtfP* was very thin and flat, which perhaps may not contain enough glucan to support the manufacture of a complex structure (Supplementary Figure S6). In contrast, the biofilm of Δ*brpL* might contain enough glucan to construct an aggregated structure. Additionally, the aggregation phenotype of Δ*brpL* was reversed in the double deletion mutant of Δ*brpL/*Δ*gtfP* (**Figure 5B**).

An interesting phenomenon observed was the presence of cavities in the center of some aggregations. As mentioned above, a similar phenomenon was also seen in *P. aeruginosa* (Ma et al., 2009). Previous works demonstrate that the formation of cell cavities is mediated by programmed cell death inside of biofilms in *P. aeruginosa* (Ma et al., 2009). Here, we also showed cavities existed in the biofilm in the layers below a cell aggregate in the biofilm of Δ*brpL* (**Figure 6A**), which might be caused by programmed cell death. In *S. sanguinis*, SpxB-mediated H_2_O_2_ induces programmed cell death (Li et al., 2016). RNA-seq data showed that the *spxB* gene was over-expressed in Δ*brpL* (Supplementary Datasheet 1), which might facilitate the accumulation of H_2_O_2_ and promote programmed cell death at the site of cavities.

Our previous work showed that *ciaR* affected biofilm formation through the arginine biosynthesis pathway (Zhu et al., 2017). The comparison of RNA-seq data from Δ*brpL* and Δ*ciaR* implied that Δ*brpL* increased biofilm formation by stimulating the expression of *ciaR*. We discovered that a PTS gene, SSA_0222, was controlled by both *brpL* and *ciaR* and was essential for the survival of *S. sanguinis* under our experimental conditions. However, although the expression of SSA_0222 was repressed in Δ*ciaR*, the Δ*ciaR* mutant was not defective in growth in BM supplemented with 1% sucrose (Zhu et al., 2017). It is still not clear whether the down-regulation of SSA_0222 contributes to reduced biofilm formation ability in Δ*ciaR*. Future efforts need to be directed into further defining the role and functionality of SSA_0222.

## Materials and Methods

### Bacterial Strains, Growth and Antibiotics

Strains used in this study are listed in Supplementary Table S1. Unless otherwise stated, strains were grown in brain heart infusion broth (BHI; Difco Inc., Detroit, MI, United States) media overnight and then diluted 100-fold into biofilm media (BM) supplemented with 1% sucrose and incubated under microaerobic conditions (6% O_2_, 7.2% CO_2_, 7.2% H_2_, and 79.6% N_2_) at 37°C using an Anoxomat^®^ system (Spiral Biotech, Norwood, MA, United States). BM supplemented with 1% sucrose was used for the growth of static biofilms and the measurement of bacterial growth (Loo et al., 2000). Kanamycin was added to a concentration of 500 μg/ml for mutant cultures. The growth of PTS mutants was tested in BM supplemented with different kinds of carbohydrate, including 1% sucrose, 1% glucose, 1% fructose, 1% lactose, and 1% galactose. OD_600_ was measured after incubation for 24 h under microaerobic conditions at 37°C.

### Mutant Construction

We have constructed Δ*gtfP*, Δ*ciaR*, and ΔSSA_0222 single gene mutants in our previous work (Ge and Xu, 2012). Based on these mutants, the *brpL* gene was deleted to construct Δ*brpL*Δ*gtfP*,Δ*brpL*Δ*ciaR*, and Δ*brpL*ΔSSA_0222 double mutants. For double mutant construction, three sets of primers were used to independently PCR amplify the 1-kb sequence of the upstream fragment of target gene, the downstream fragment of target gene and the *erm* gene for erythromycin resistance. Primers listed in Supplementary Table S2. The three fragments were combined by a second round of PCR. The final recombinant PCR product was transformed into *S. sanguinis* SK36 single mutants. Double mutants were selected by erythromycin resistance and confirmed by PCR analysis. BHI medium was used in all processes of mutant construction.

### CV Staining Assay

Overnight cultures were diluted 1:100 into BM supplemented with 1% sucrose in a 96-well microtiter plate (Falcon 3911). After incubation at 37°C for 24 h under microaerobic conditions, the supernatant was gently removed by pipetting. Biofilms were washed once with distilled water and stained by the addition of 0.4% CV for 30 min at room temperature. CV was then gently removed by pipetting. Biofilms were washed twice with distilled water, solubilized in 30% acetic acid and measured at *A*_560_ as described previously (Ma et al., 2006).

### Biofilm Attachment Assay

Biofilms were tested by a protocol similar to the CV staining assay. The differences were: CV and water were injected into 96 wells at the CV staining step and washing step, respectively, by using a Caliper Sciclone G3 liquid handling robot (PerkinElmer, United States) with different speeds of injection.

### Static Biofilm Assay

Static biofilms were grown in 4-chambered glass coverslip wells (Chambered Coverglass, Thermo Scientific) in BM supplemented with 1% sucrose at 37°C under micro-aerobic conditions for 24 h. The supernatant was discarded and biofilms were washed with PBS. For testing biomass, biofilms were stained with a live/dead staining kit (Invitrogen, United States) in darkness for 10 min. STYO9 (green signal) stained live cells and PI (red signal) stained dead cells and eDNA. Two methods were used to stain cells and polysaccharide. In **Figure 4A**, cells (red signal) were marked with Hexidium iodide (HI) (Invitrogen, United States) at 4.7 μM and polysaccharide containing α-(1, 3) or α-(1, 6) linked mannosyl units (green signal) was stained by 100 μg/mL of *Hippeastrum* hybrid lectin (HHA)-FITC (EY Labs, United States) (Ma et al., 2009). Biofilms were stained in darkness for 2 h. In **Figure 4B**, biofilms were cultured in BM supplemented with 1% sucrose and 10 μM of Alexa Fluor 647-labeled dextran conjugate (Invitrogen, United States) for 24 h in darkness. The fluorescently labeled dextran was used as an acceptor and was incorporated into newly formed glucan by Gtfs (Koo et al., 2010). Then the supernatant was discarded and biofilms were stained by 5 μM of SYTO 9 (Invitrogen, United States) for 10 min. The fluorescent images were acquired with a Zeiss LSM710 CLSM (Zeiss, Germany) and quantified by COMSTAT in Matlab (Heydorn et al., 2000). Three images of each sample were quantified to calculate the means and standard deviations.

### Growth Curve Measurement

Strains were cultured in BM supplemented with 1% sucrose in 96-well plates with continuous shaking and growth was monitored every 30 min at 600 nm with a Synergy H1 Hybrid Reader (BioTek, United States). The microaerobic conditions (6% O_2_, 6% CO_2_) were maintained by injection of CO_2_ and N_2_ to maintain CO_2_/O_2_ set concentrations (BioTek, United States). Three replicates were examined to calculate the means and standard deviations.

### Biofilms Treated by DNase I

Biofilms were grown in BM supplemented with 1% sucrose for 24 h and then supernatant was discarded by pipetting. PBS buffer or PBS supplemented with 100 U/mL of DNase I was added to the wells. Biofilms were treated by DNase I for 2 h under microaerobic condition at 37°C and then biomass was measured using CV staining. Two kinds of DNase I (QIAGEN, catalog number: 79254; Thermo Scientific, catalog number: FEREN0525) were used in this assay and results were the same.

### Scanning Electronic Microscopy (SEM) Analysis of Biofilm

Biofilms were grown as previously described, on the surface of cover glasses (Fisher Scientific, catalog number: 083110-9). The 1-day biofilms were washed twice with PBS in Petri dishes and fixed with 2% glutaraldehyde overnight. Following dehydration through a graded series of ethanol, the cover glasses were air dried and sputter coated with gold. Samples were then scoped by a SEM machine (Zeiss EVO 50 XVP, Jena, Germany).

### Maximum Tolerance Concentration of Strains to Ampicillin

Biofilms were grown in BM for 24 h and then supernatant discarded by pipetting. Biofilms were treated with different concentrations of ampicillin for 2 h in microaerobic condition at 37°C. After ampicillin treatment, supernatant was discarded and cells were resuspended in PBS by pipetting. Bacteria cultures were centrifuged, resuspended in PBS and diluted 100-fold into fresh BHI in 96-well plates. After overnight culturing, if cells could survive after ampicillin treatment, they would grow in fresh BHI and let medium turbid. Failure to grow would result is a completely clear media. Three replicates were examined to get the results.

The tolerance of cells to ampicillin in planktonic form was similar to biofilm cell tolerance. Cells were cultured overnight in BHI. OD_600_ of planktonic cells was tested by a Synergy H1 Hybrid Reader (BioTek, United States). As the biofilm biomass of Δ*brpL* was nearly two times higher than WT, we collected planktonic Δ*brpL* cells of biomass two times more than that of WT. These planktonic cells were treated with ampicillin using the same processes as mentioned above.

### The Measurement of WIG and WSG

Water-insoluble glucan and WSG was measured as previously described (Liu et al., 2017; Zhu et al., 2017). Biofilms were grown in BM for 24 h in 24-well plates. The supernatant was then removed and biofilms were resuspended in 1 mL of distilled water. One-half mL of cell suspension was prepared for the determination of total protein concentration. Another 500 μL of bacterial suspension was centrifuged. The supernatant was prepared for the measurement of WSG. The sediment was dissolved in the same volume of 1 N NaOH for 3 h and centrifuged. The supernatants were precipitated by three volumes of isopropanol for 1 day at -20°C. The precipitates obtained by centrifugation were then air dried and dissolved in 250 μL of ddH2O for WSG or 1 N NaOH for WIG. The amount of glucans in each fraction was quantified by the phenol-sulfuric acid method as previously described (Decker et al., 2011). Glucose was used as a reference carbohydrate to generate a standard curve. The concentrations of WIG and WSG were normalized by total protein concentration in the biofilm. Three replicates were examined to calculate the means and standard deviations.

### The Measurement of Protein Concentration

Cells were harvested and resuspended in lysis buffer (Tris pH7.4 50 mM, NaCl 150 mM, glycerol 10%, NP-40 1%, SDS 0.1%). Cell suspensions were incubated on ice for 30 min and then lysed by mechanical disruption using FastPrep lysing matrix B (Qbiogene, Irvine, CA, United States). The protein concentration of cell lysate was measured by following the standard protocol of Pierce^TM^ BCA Protein Assay Kit (Thermo Scientific). Four replicates were analyzed to calculate the means and standard deviations.

### qRT-PCR Assay

The WT and mutants were cultured in BHI overnight and then diluted into fresh BHI and grown for 3 h in microaerobic conditions at 37°C. Samples were collected, treated with RNA protect bacteria reagent (Qiagen, Valencia, CA, United States) for 5 min to stabilize RNA and stored at -80°C. Cells were lysed by mechanical disruption using FastPrep lysing matrix B (Qbiogene, Irvine, CA, United States). Total RNA was treated with DNase I (Qiagen) and prepared using RNA easy mini kits (Qiagen) according to the manufacturer’s instructions. RNA extraction was performed as described below for the RNA-seq assay. Reverse transcription followed the standard procedure provided with the SuperScript^TM^ III Reverse Transcriptase Kit (Qiagen). The cDNA was used as the template, combined with 2X SYBR Green PCR Master Mix (Qiagen) and the q-PCR primers were shown in Supplementary Table S2. Gene expression in mutants is relative to that in WT. The housekeeping gene *gyrA* was used as a normalization control (Ge et al., 2016). Three replicates were analyzed to calculate the means and standard deviations.

### RNA-seq and Data Analysis

The WT and Δ*brpL* were cultured in BHI medium overnight and then diluted into fresh BHI medium. After incubation in microaerobic conditions at 37°C for 3 h, samples were collected, treated with RNA protect bacteria reagent (Qiagen, Valencia, CA, United States) for 5 min to stabilize RNA and stored at -80°C. Cells were lysed by mechanical disruption using FastPrep lysing matrix B (Qbiogene, Irvine, CA, United States). Total RNA was treated with DNase I (Qiagen) and prepared using RNA easy mini kits (Qiagen) according to the manufacturer’s instructions. Ribo-Zero Magnetic Kit for Bacteria (Illumina) was used to deplete ribosomal RNA from 2 μg of total RNA. NEBNext Ultra Directional RNA Library Prep Kit for Illumina (New England BioLabs) was used for the following RNA-seq library preparation according to the manufacturer’s protocol. Library sequencing was performed by the Nucleic Acids Research Facilities at Virginia Commonwealth University using an Illumina HiSeq2000 instrument. The raw RNA sequencing data are available in the NCBI Gene Expression Omnibus (GEO)^2^ under the accession number: GSE110307. Reads obtained from RNA-seq were aligned against the *S. sanguinis* SK36 genome using EDGE-pro (Magoc et al., 2013). Differential gene expression was analyzed by DESeq2 (Love et al., 2014). *P*-values shown in RNA-seq data are adjusted *p*-values generated by DESeq2. Four replicates were performed for analysis. The RNA-seq data of Δ*ciaR* was reanalyzed by EDGE-pro and DESeq2 (Magoc et al., 2013; Love et al., 2014).

Based on the knowledge of KEGG database (Kanehisa et al., 2004), the regulated genes (fold change ≥ 1.5 or ≤0.667 and *p*-value ≤ 0.05) in Δ*brpL* were classified into different function groups. We wrote a script, named RPAS, in Matlab software to count the number of genes involved in each group (Supplementary Script of Matlab).

### Statistical Analysis

All data were obtained from at least three biological replicates. Student’s *t*-test was applied to analyze data on biofilm assay, COMSTAT results, qRT-PCR, cell growth and the production of WIG and WSG.

### Data Availability

The datasets generated during and/or analyzed during the current study are available from the corresponding author upon reasonable request.

## Author Contributions

BZ and PX conceived and designed this study. BZ carried out all the experiments with the assistance of LS and LM. BZ, XK, and PX analyzed the data and wrote this manuscript. All authors reviewed and discussed the manuscript.

## Conflict of Interest Statement

The authors declare that the research was conducted in the absence of any commercial or financial relationships that could be construed as a potential conflict of interest.

## Footnotes

1 http://smart.embl.de/

2 https://www.ncbi.nlm.nih.gov/geo/
