# Supplementary material for: A Novel Regulator Modulates Glucan Production, Cell Aggregation and Biofilm Formation in Streptococcus sanguinis SK36
Source: Front Microbiol. 2018 May 29;9:1154. doi: 10.3389/fmicb.2018.01154 (PMC5987052; doi:10.3389/fmicb.2018.01154)
Supplement: Supplementary file 1 [file Presentation_1.PDF]

A novel regulator modulates glucan production, cell aggregation and biofilm formation in *Streptococcus sanguinis* SK36

Bin Zhu[1], Lei Song[2], Xiangzhen Kong[1], Lorna C Macleod[1] and Ping Xu[1, 2, 3,*]

Supporting information

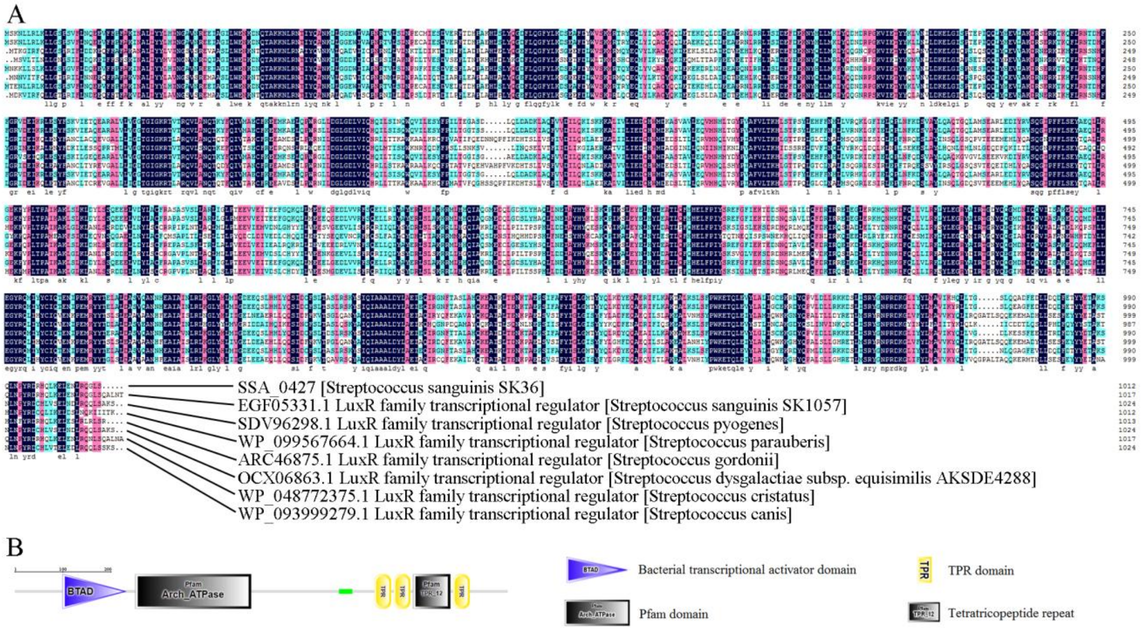

Fig. S1 The protein sequence alignment of BrpL (A) and its secondary structures predicted by SMART (B).

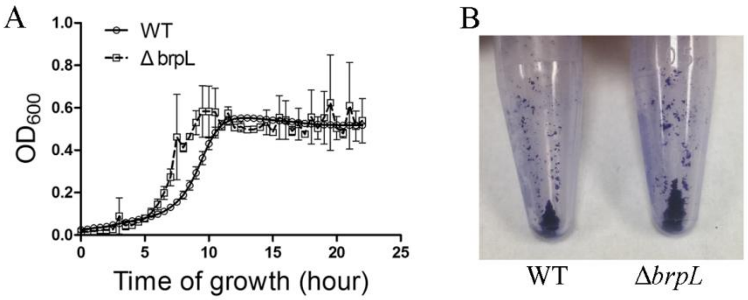

Fig. S2 The growth of Δ*brpL*. WT and Δ*brpL* were cultured in BM supplied with 1% sucrose. (A) Growth curves of WT and Δ*brpL* were recorded by a plate reader. (B) Cells were cultured for 7.5 hours, harvested and stained by CV. The digital picture was shown to exhibit the biomass of strains.

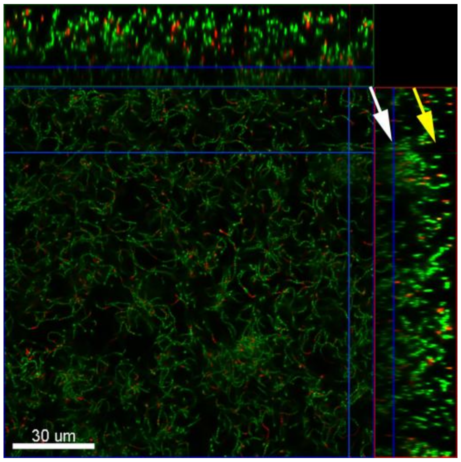

Fig. S3 Cell aggregation and cell cavities in the biofilm of Δ*brpL*. The biofilm of Δ*brpL* was cultured in a 4-well chamber for 24 hours, stained by SYTO 9 (green) / PI (red) and observed by CLSM. The white arrow indicates a group of cell aggregation. The yellow arrow points to a cell cavity at the bottom of cell aggregation.

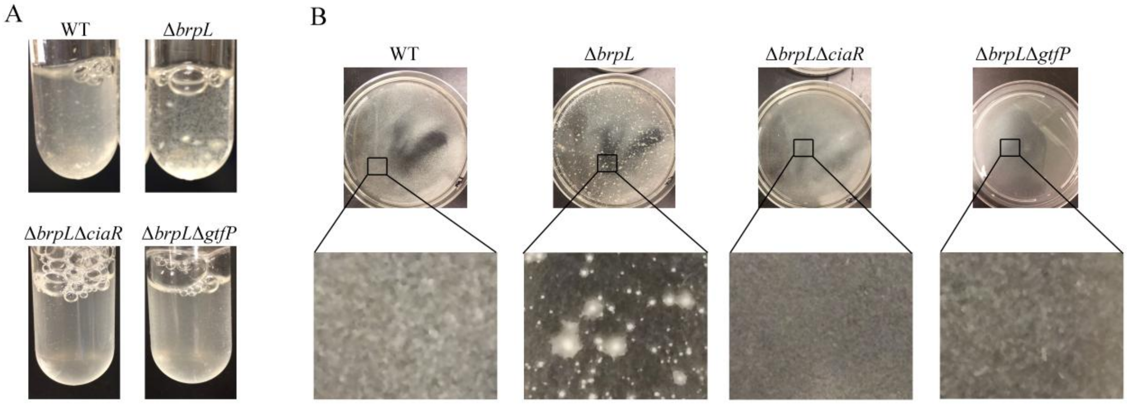

Fig. S4 Cell aggregation in 14 mL tubes and on bacteriological petri dishes. (A) Cells were cultured in BM supplied with 1% sucrose in shaking conditions for 5 hours by using 14 mL tubes. (B) Cells were statically incubated in BM supplied with 1% sucrose in bacteriological petri dishes for 24 hours. The digital pictures showed the morphology of biofilms on the surface of dishes.

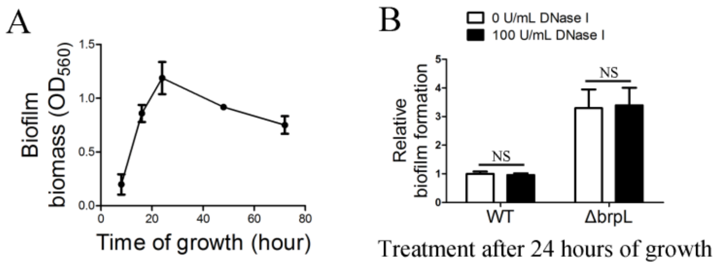

Fig. S5 The effect of DNase I treatment on biofilm formation. (A) Biofilm biomass was recorded by CV staining after different time of incubation. (B) Biofilms were grown in BM supplied with 1% sucrose for 24 hours and then treated with 100 U / mL of DNase I for 2 hours. Biofilm biomass was tested by CV staining.

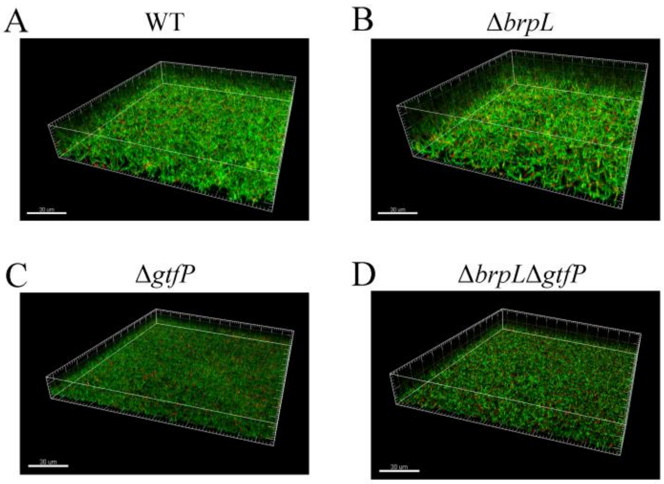

Fig. S6 The CLSM images of biofilms. Biofilms were cultured in 4-well chambers for 24 hours, stained by SYOT9/PI and then observed by CLSM. Images of biofilm 3D structures were shown.

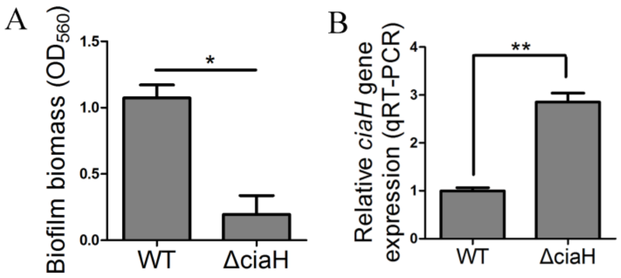

Fig. S7 The expression of *ciaH* gene in Δ*brpL*. (A) The biofilm formation ability of Δ*ciaH* was assayed by CV staining. (B) qRT-PCR was performed to examine the expression of *ciaH* in Δ*brpL*.

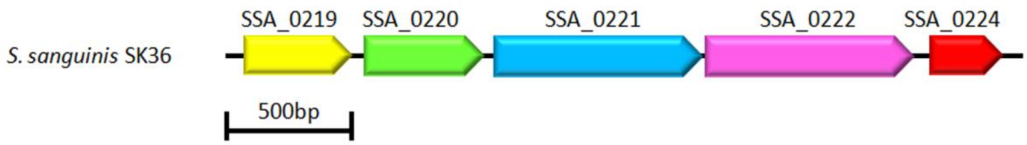

Fig. S8 The schematic of the activated PTS genes cluster in Δ*brpL*.

Table S1 Strains used in this study.

**Strain or plasmid**	**Genotype and/or relevant characteristics**	**Source or reference**
S. sanguinis strains		
SK36	Wild type, Human plaque isolate	(Kilian and Holmgren, 1981)
Δ*brpL*	SK36, insert-deletion of *brpL* gene, Km^r	(Ge and Xu, 2012)
Δ*gtfP*	SK36, insert-deletion of *gtfP* gene, Km^r	(Ge and Xu, 2012)
Δ*ciaR*	SK36, insert-deletion of *ciaR* gene, Km^r	(Ge and Xu, 2012)
Δ*ciaH*	SK36, insert-deletion of *ciaH* gene, Km^r	(Ge and Xu, 2012)
ΔSSA_0222	SK36, insert-deletion of SSA_0222 gene, Km^r	(Ge and Xu, 2012)
Δ*brpL* Δ*gtfP*	SK36, insert-deletion of *gtfP* gene based on Δ*brpL*, Km^r Erm^r	This study
Δ*brpL* Δ*ciaR*	SK36, insert-deletion of *ciaR* gene based on Δ*brpL*, Km^r Erm^r	This study
Δ*brpL* ΔSSA_0222	SK36, insert-deletion of SSA_0222 gene based on Δ*brpL*, Km^r Erm^r	This study

Table S2 primers used in experiments

name	Sequence (5’-3’)	Application
F1- brpL	AAGTGGAGAACGAAGGCTG	Double gene mutant construction
R1- brpL	TGTAATCACTCCTTCTCACTATTTAGTGG AGATAGTAAAGCAGAGCATT	Double gene mutant construction

F2-erm	TAAATAGTGAGAAGGAGTGATTACATGAACAA	Double gene mutant construction
R2-erm	TTATTTCCTCCCGTTAAATAATAG	Double gene mutant construction
F3- brpL	CTATTATTTAACGGGAGGAAATAAGCATCAACTGCTGACTGGCA	Double gene mutant construction
R3- brpL	GCTTCCTTGGCATCTTGG	Double gene mutant construction
F-qpcr-gyrA	AGCTGATTGCCTTGATTGCAGAC	q-PCR of *gyrA* gene
R-qpcr-gyrA	ATCCGCAAATTTACGCTTGACCT	q-PCR of *gyrA* gene
F-qpcr-gtfP	GCCCAAATTCTCAACCGTTAC	q-PCR of *gtfP* gene
R-qpcr-gtfP	ATCTTGCCCTTGACTTGGTAG	q-PCR of *gtfP* gene
F-qpcr-argR	GCGTCATTGTAACCCAGTCC	q-PCR of *argR* gene
R-qpcr-argR	GGATGGTGCTATGCTATTGATG	q-PCR of *argR* gene
F-qpcr-argC	GCCTTGATTCCCTTGCTG	q-PCR of *argC* gene
R-qpcr-argC	TGGATGCGGCTGGATT	q-PCR of *argC* gene
F-qpcr-argJ	GCTATCGGCTATGCGGG	q-PCR of *argJ* gene
R-qpcr-argJ	CATCACTTGTTTCCTCGGG	q-PCR of *argJ* gene
F-qpcr-argB	CAGGTGTCATCAAGGGTGGTA	q-PCR of *argB* gene
R-qpcr-argB	CTATCAAGGTGCCCGTCAA	q-PCR of *argB* gene
F-qpcr-argH	TGAGTTTGATGTGCGTAATGAA	q-PCR of *argH* gene
R-qpcr-argH	GGAGCGAGCCGTATGAAG	q-PCR of *argH* gene
F-qpcr-argG	GGTGTTGGGCGGATTG	q-PCR of *argG* gene
R-qpcr-argG	CGATTTCCTTGTGGGCAG	q-PCR of *argG* gene
F-qpcr-ciaR	AAAGGACGGTTTCCAAGTGC	q-PCR of *ciaR* gene
R-qpcr-ciaR	TTACCAGCGCGTTTCAAGAG	q-PCR of *ciaR* gene
F-qpcr-ciaH	ATGAGTGTCAAGCCTCTGCT	q-PCR of *ciaH* gene
R-qpcr-ciaH	CGAAAGAGCGTCTCTAAGCG	q-PCR of *ciaH* gene
F-qpcr-brpL	GGATAAGGCCAGTGTTGCTG	q-PCR of *brpL* gene
R-qpcr-brpL	GTGCTCAGATGCTTGGTCAG	q-PCR of *brpL* gene
F-qpcr-brpT	CTATCGCGCCAGGCTATCTA	q-PCR of *brpT* gene

R-qpcr-brpT	TGGGCAAAGAAACAGAACGG	q-PCR of *brpT* gene
F-qpcr-SSA_0222	GGCGAAGCTTTCCTGAAGTT	q-PCR of SSA_0222 gene
R-qpcr-SSA_0222	TCAAAGATGCCAAGCGCTAC	q-PCR of SSA_0222 gene

Ge, X., and Xu, P. (2012). Genome-wide gene deletions in Streptococcus sanguinis by high throughput PCR. *J Vis Exp* (69). doi: 10.3791/4356.

Kilian, M., and Holmgren, K. (1981). Ecology and nature of immunoglobulin A1 protease-producing streptococci in the human oral cavity and pharynx. *Infect Immun* 31(3)**,** 868-873.
