# Supplementary material for: A Novel Regulator Modulates Glucan Production, Cell Aggregation and Biofilm Formation in Streptococcus sanguinis SK36
Source: Front Microbiol. 2018 May 29;9:1154. doi: 10.3389/fmicb.2018.01154 (PMC5987052; doi:10.3389/fmicb.2018.01154)
Supplement: Supplementary file 2 [file Presentation_2.ZIP › RNA_seq_KEGG_pathway_analysis/Instructions.docx]

1. Input RNA-seq data in an excel file and name it as ‘data.xlsx’ (see example)

2. Put these three files in the same folder:

data.xlsx

KEGG pathway_gene.xlsx

RPAS.m

3. Run RPAS.m in Matlab. An output file “Results.xlsx” will be generated.

4. Numbers of regulated genes are shown in sheet 1 of Results.xlsx.

5. If you are interested in a certain pathway, you can find the ko number of this pathway in the first column of sheet 1. Search this ko number in sheet 2. There will be a gene list involving all of the regulated genes in the pathway.

6. If you want to discover the functions of these genes, you can go to the website of KEGG mapper (<http://www.genome.jp/kegg/tool/map_pathway1.html>).

Click “Search&Color pathway” (yellow arrow), input “ssa” (red arrow), paste gene list and color list from sheet 2 of Results.xlsx to the input box of the website (blue arrow). Click “Exec” (green arrow).


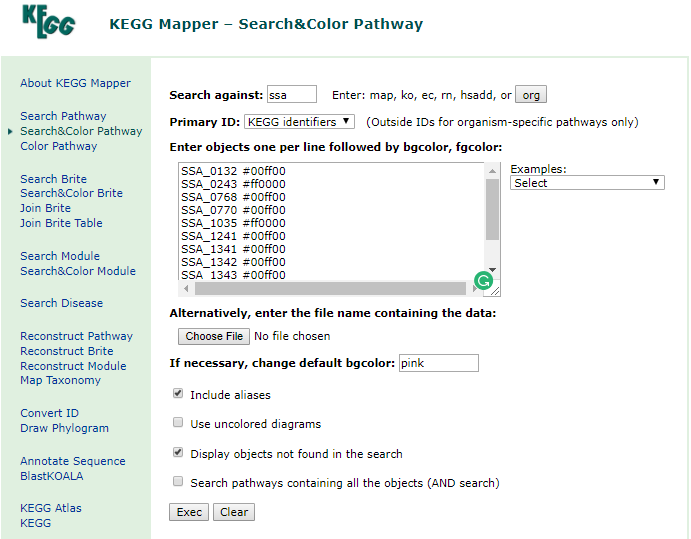


Edited by Bin Zhu at Virginia Commonwealth University, email: bzhu@vcu.edu
